# Robust R-peak detection in an electrocardiogram with stationary wavelet transformation and separable convolution

**DOI:** 10.1038/s41598-022-19495-9

**Published:** 2022-11-16

**Authors:** Donghwan Yun, Hyung-Chul Lee, Chul-Woo Jung, Soonil Kwon, So-Ryoung Lee, Kwangsoo Kim, Yon Su Kim, Seung Seok Han

**Affiliations:** 1 Department of Biomedical Sciences, Seoul National University College of Medicine, Seoul, Korea; 2 Department of Internal Medicine, Seoul National University College of Medicine, Seoul, Korea; 3 Department of Anesthesiology and Pain Medicine, Seoul National University College of Medicine and Seoul National University Hospital, Seoul, Korea; 4 Division of Cardiology, Department of Internal Medicine, Seoul National University Hospital, Seoul, Korea; 5 Transdisciplinary Department of Medicine and Advanced Technology, Seoul National University Hospital, Seoul, Korea

**Keywords:** Cardiology, Health care

## Abstract

R-peak detection is an essential step in analyzing electrocardiograms (ECGs). Previous deep learning models reported their performance primarily in a single database, and some models did not perform at the highest levels when applied to a database different from the testing database. To achieve high performances in cross-database validations, we developed a novel deep learning model for R-peak detection using stationary wavelet transform (SWT) and separable convolution. Three databases (i.e., the MIT-BIH Arrhythmia [MIT-BIH], the Institute of Cardiological Technics [INCART], and the QT) were used in both the training and testing models, and the MIT-BIH ST Change (MIT-BIH-ST), European ST-T, TELE and MIT-BIH Noise Stress Test (MIT-BIH-NST) databases were further used for testing. The detail coefficient of level 4 decomposition by SWT and the first derivative from filtered ECGs were used for model inputs, and the interval of 150 ms centered at marked peaks was used for labels. Separable convolution with atrous spatial pyramidal pooling was selected as the model’s architecture, and noise-augmented waveforms of 5.69 s duration (2048 size in 360 Hz) were used in training. The model performance was evaluated using cross-database validation. The F1 scores of the peak detection model were 0.9994, 0.9985, and 0.9999 in the MIT-BIH, INCART, and QT databases, respectively. When the above three databases were pooled, the F1 scores were 0.9993 for fivefold cross-validation and 0.9991 for cross-database validation. The model performance remained high for MIT-BIH-ST, European ST-T, and TELE, with F1 scores of 0.9995, 0.9988, and 0.9790, respectively. The model performance when trained by severe noise augmentation increased for the MIT-BIH-NST database (F1 scores from 0.9504 to 0.9759) and decreased for the MIT-BIH database (F1 scores from 0.9994 to 0.9991). The present SWT and separable convolution-based model for R-peak detection yields a high performance even for cross-database validations.

## Introduction

Detecting R-peaks in electrocardiograms (ECGs) is an initial and essential step to identify components and arrhythmias. Classic detection models, such as Pan-Tompkins^1^, Hamilton^2^, and Christov^3^, consist of filtering noise, enhancing peaks, and adaptive thresholding, which display favorable performances in open source databases. Further strategies may be applied during the peak enhancement step, such as waveform derivatives^4^, Hilbert transform^5^, discrete wavelet transform (DWT)^6^, and stationary wavelet transform (SWT)^7^. These peak enhancement methods effectively extract peak information from the waveforms, although the downstream pipeline of adaptive thresholding depends on a few hyperparameters, and there is room for improvement in the model performance by hyperparameter tuning.

Recently, deep learning methods, such as convolutional neural network (CNN)^8–12^ and long-short term memory of recurrent neural network (RNN)^13,14^, have been used for peak detection. The layers in deep learning models play a role in peak enhancement and adaptive thresholding during detection. Although deep learning models have achieved good success for peak detection, certain limitations have been noted, particularly in cross-database validation^15,16^. Most open source databases have a relatively modest number of patients and a unique shape of peaks, which might lead to overfitting while training and low performances in other databases.

To overcome the above limitations, we used peak enhancement methods in the classic peak detection models and transformed waveforms during the model training. This was because of the hypothesis that peak enhancement may generalize various peak shapes and alleviate the intrinsic differences among the ECG databases. We adopted SWT as the peak enhancement method, which maintained the original length of the waveforms after transformation and showed stable performance in several databases and with different adaptive thresholding methods^7,17^.

We also used separable convolution^18^ followed by atrous spatial pyramid pooling (ASPP)^19^, which achieved a good performance in segmenting and classifying the images but has not yet been explored for ECG peak detection. Separable convolution effectively extracts important features with fewer parameters than a classic convolution and was a follow-on to atrous convolution (also known as dilated convolution), making it possible to obtain wide receptive fields. Peak enhancement based on SWT and adaptive thresholding by a deep learning model provides an efficient encoder-decoder structure, which yields high performance in cross-database validation.

## Methods

### Database

For the training and testing processes, we used open source databases, such as the MIT-BIH Arrhythmia (MIT-BIH), the Institute of Cardiological Technics (INCART), and the QT^20–22^. These databases had 48 half-hour two-lead ECGs with a sampling rate of 360 Hz, 75 half-hour twelve-lead ECGs with a sampling rate of 257 Hz, and 82 fifteen-minute two-lead ECGs with a sampling rate of 250 Hz. We excluded the ECG areas with ventricular flutter rhythm in the 207 record of the MIT-BIH because this arrhythmia was only present in the MIT-BIH database and was not properly trained in a cross-database as was done in a similar previous study^13^. The ECG waveforms of lead II from the databases were used throughout the analysis.

The MIT-BIH ST Change (MIT-BIH-ST)^23^, European ST-T^24^, and TELE^25^ databases were used for validation. The MIT-BIH-ST included 28 ECG recordings with varying lengths that were recorded during exercise stress test and had a transient depression or elevation of the ST segment. European ST-T consisted of 90 ambulatory ECG recordings from 79 subjects with varying lengths, and baseline ST segment displacement resulted from hypertension, ventricular dyskinesia, effects of medication, and others. TELE was recorded by dry metal electrodes and contained 250 ECG recordings with short duration less than 5 s.

The MIT-BIH-NST databases^26^ was used for exploring the effect of noise parameters, The records in the MIT-BIH-NST were obtained by adding electrode motion artifacts to two clean records from the MIT-BIH. Signal-to-noise ratios (SNRs) in the range of 24 dB to -6 dB were included in the MIT-BIH-NST.

### Preprocessing step

ECG waveforms of lead II were resampled to 360 Hz by the fast Fourier transformation method. Because we used multiple databases that contained various types of noise, we adopted noise filtering methods to remove unwanted features and to focus on peak features. After applying DWT to attenuate the baseline wandering by a decomposition down to level 9^27^, a lowpass filter with a cutoff of 40 Hz was used to remove powerline noise.

**Figure 1:**
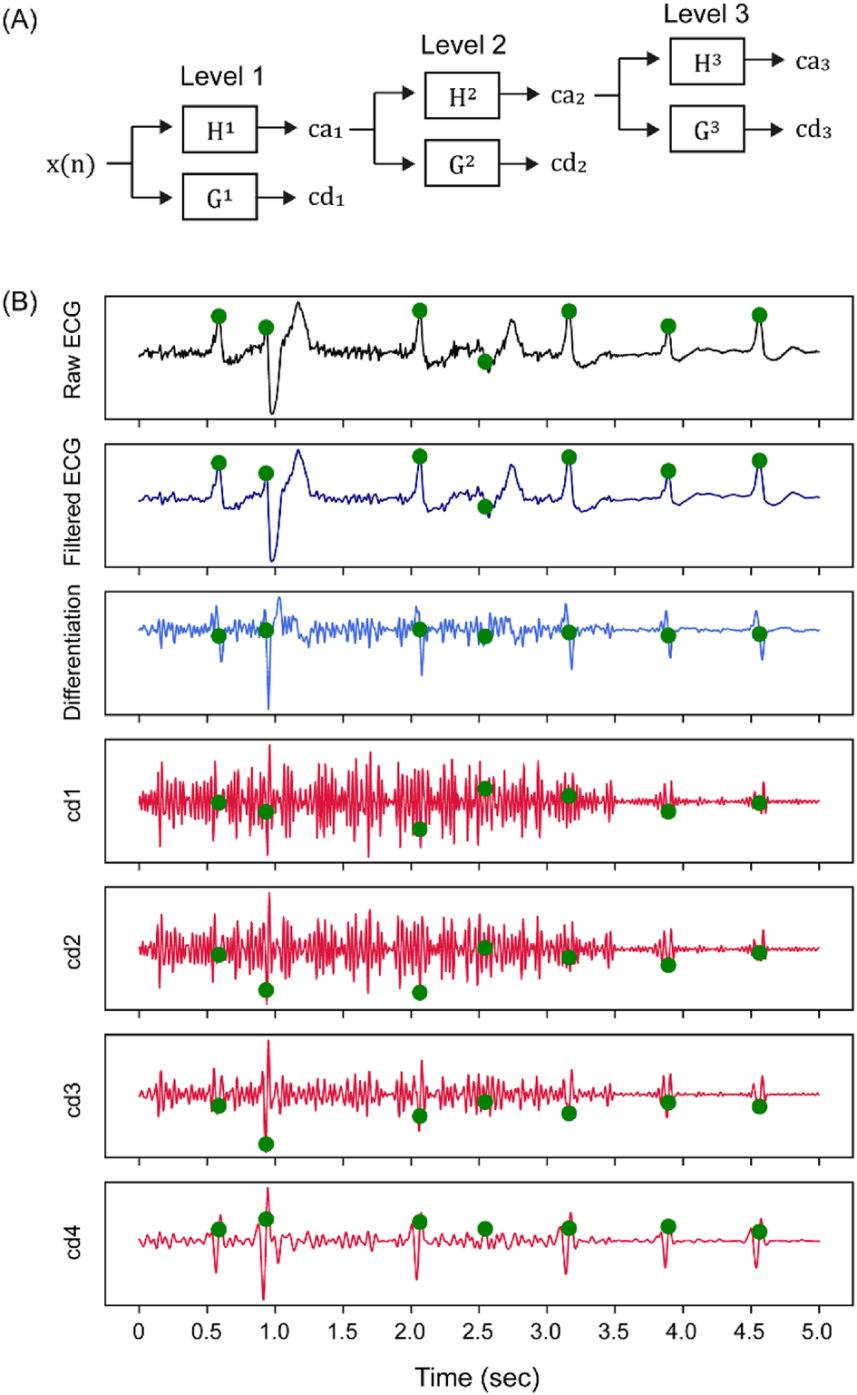
The SWT process according to the decomposition levels. The ECG record was derived from patient 114 of the MIT-BIH database. (**A**) Tree-structured decomposition process of SWT. (**B**) ECG waveforms after filtering, differentiation and the detail coefficients of SWT. Unwanted features of original waveforms are attenuated after a level 4 decomposition of SWT. Green dots represent marked peak positions. *cd* detail coefficient of SWT, *G* lowpass filter, *H* highpass filter.

As a peak enhancement method, we applied SWT in the preprocessing step. SWT has a tree-structured decomposition similar to DWT (Fig. 1A) but the major difference is the shift invariance. Any level of decomposition with SWT has the same wavelet length, which is suitable for peak localizations. In the level $k$ decomposition with SWT, the level-adaptive size-varying highpass filter (${H}^{k}$) and lowpass filter ${G}^{k}$ perform a convolution on the input signal and yield approximate coefficients ${ca}_{k}$ and detail coefficients ${cd}_{k}$, respectively. Highpass filter ${H}^{k+1}$ and lowpass filter ${G}^{k+1}$ are upsampled by a factor of two from their previous stage (i.e., ${H}^{k}$ and ${G}^{k}$), and therefore, the coefficients ${ca}_{k+1}$ and ${cd}_{k+1}$ have the same length as the original signal and their previous coefficients ${ca}_{k}$ and ${cd}_{k}$.

The signal energy of ECG is usually concentrated in a frequency ranging from 3 to 40 Hz^28^, and we used the detail coefficients of SWT in a level four decomposition with wavelet Symlet 4 according to the energy analysis of a previous study (Fig. 1B)^7^. Despite an effective feature extraction of SWT, high amplitude areas of SWT slightly lead the true peak positions, and we also used the first derivative of the waveforms as peak enhancement methods to make the model find accurate peak positions. Finally, these two waveforms after peak enhancement were normalized by dividing them by 16 times the root mean squares of each waveform, and their absolute values rarely exceeded 1. These waveforms were used as 1-D features with two channels.

To formulate peak detection as a segmentation problem, binary labeling was used, and areas of 150 ms at the center of peaks were filled with ones, while the rest were labeled with zeros. These arrays were prepared as targets and used to train the model.

### Model architecture

The model architecture was inspired by DeepLabv3 + ^19^, which used Xception with a separable convolution^18^ followed by an atrous spatial pyramidal pooling (ASPP). The separable convolution consisted of a depthwise convolution (i.e., a spatial convolution performed independently over each channel) and a pointwise convolution (i.e., 1 × 1 convolution) and showed an efficient image classification performance with fewer hyperparameters when compared with a conventional convolution. ASPP used a dilated convolution with variable kernel sizes, which contributed to wide multiscale receptive fields. Although DeepLabv3 + achieved the state-of-the-art in image segmentation field, it was primarily developed for 2-D input and was not able to be directly applied for 1-D input.

**Figure 2:**
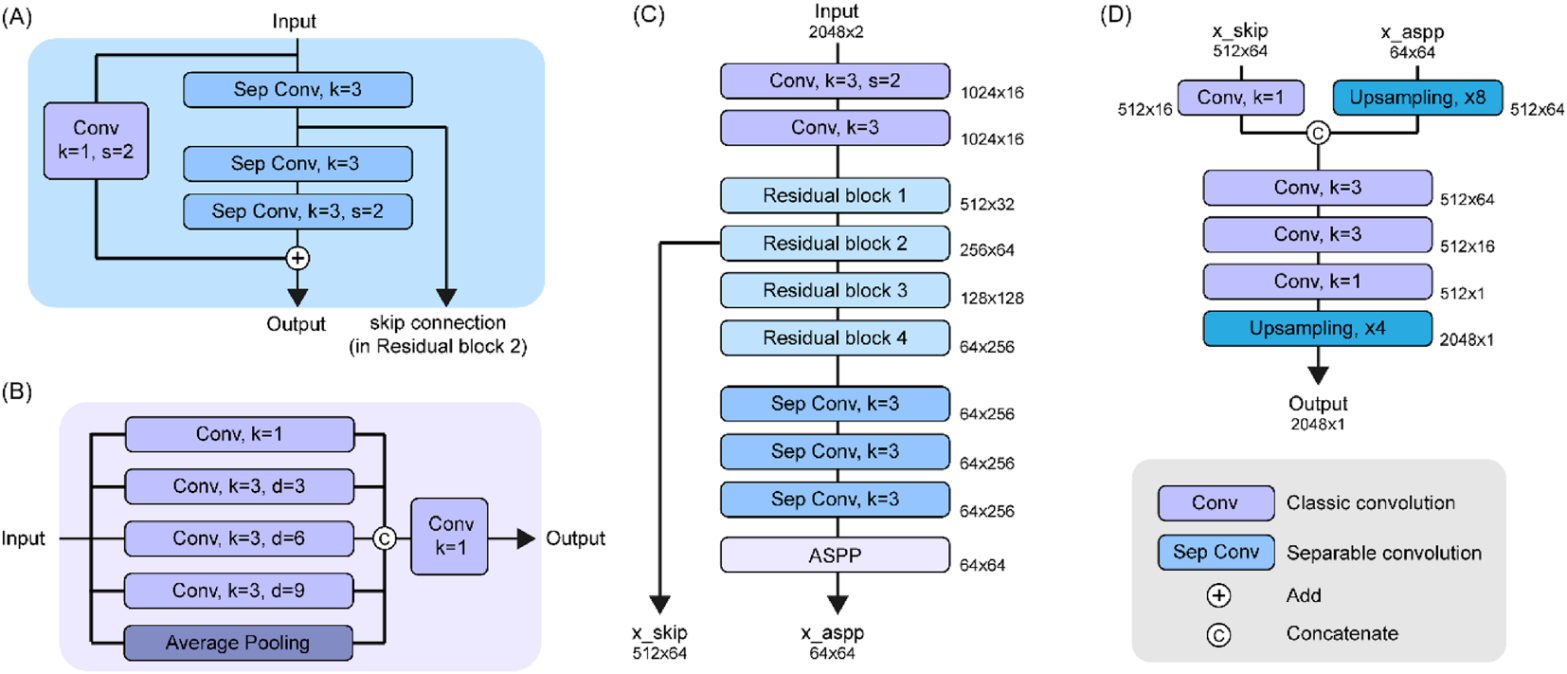
Detailed architecture of the proposed encoder-decoder model. A batch normalization and ELU are followed by each classic or separable convolution layer, while a residual block starts with ELU for a preactivation. (**A**) The structure of the residual block. (**B**) The structure of atrous spatial pyramidal pooling. (**C**) The structure of the encoder. Tensor shapes without batch sizes are summarized as the sequence length × the number of channels. (**D**) The structure of the encoder and its output. *ASPP* atrous spatial pyramidal pooling, *Conv* classic convolution, *d* dilatation rate, *k* kernel size, *s* stride size, *Sep Conv* separable convolution.

Our proposed CNN model uses residual blocks with preactivation^29^ and three sequential separable convolutional 1-D layers. The encoder downsampled the model input 5 times by a conventional convolution and residual blocks and ended with ASPP. The decoder performed an upsampling to the original length by merging ASPP and the skip connection from the encoder. All 1-D filters except 1 × 1 convolution and ASPP had a kernel size of 3, and ELU activation^30^ was used. The detailed model structure is summarized in Fig. 2.

There are several important differences between the original DeepLabv3 + architecture and ours. First, the number of hyperparameters was considerably decreased and the mid-flow of the encoder was omitted, because a peak detection (i.e., binary classification) did not require as many hyperparameters as a multiclass classification. Second, we performed one more downsampling in the encoder. The distance between two peak positions cannot be less than 200 ms of the cardiac refractory period, and the model needs to compress peak information rather than restore it to a high-resolution segmentation in the peak detection problem. Kernel sizes in ASPP were also reduced by half (from [1, 6, 12, 18] to [1, 3, 6, 9]) as the compression rate doubled. Finally, the method of global average pooling in ASPP was modified. Although the original ASPP dealt with a single image, our model needs to receive time-series data, and global average pooling in the whole sequence of the waveform may not reflect the temporary features. In this regard, the current model calculated the averages of the features in units of 2048 in size.

### Training step

**Figure 3:**
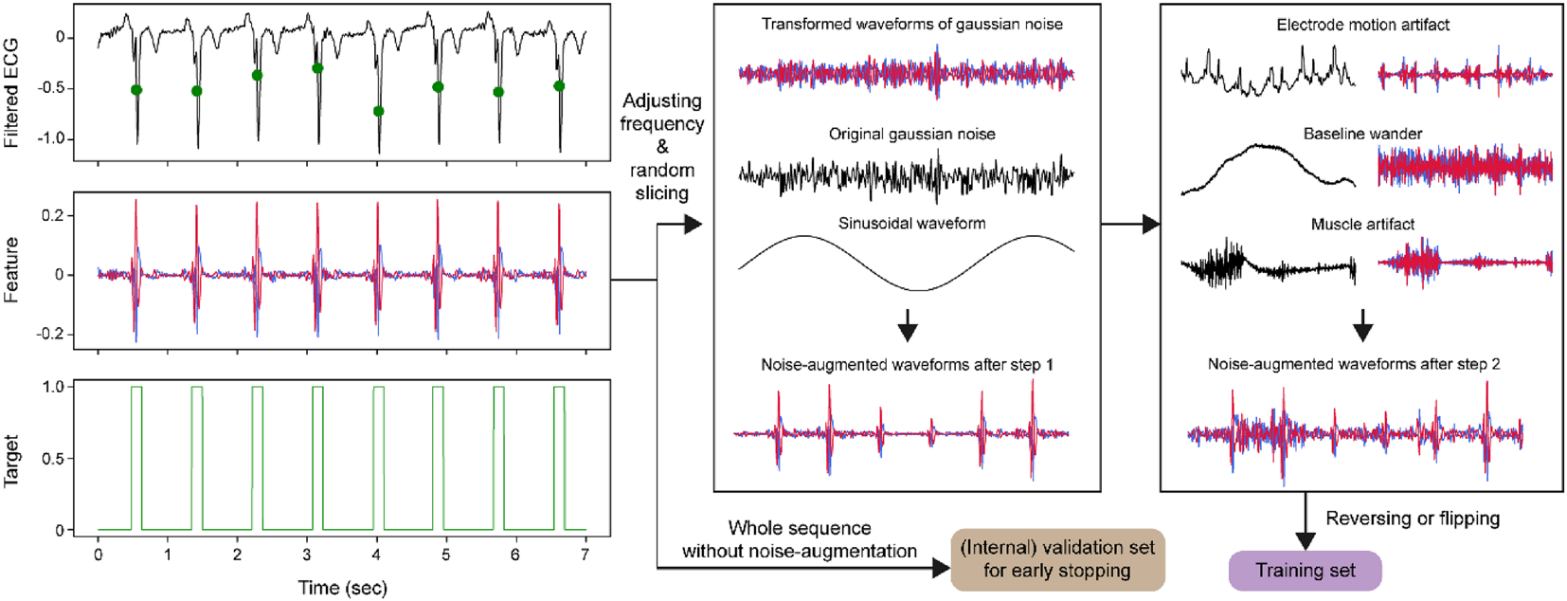
The schematic plot of a noise-augmented waveform generation. The ECG record was derived from patient I54 in the INCART database. Peak positions with 150 ms duration are filled as one and prepared for the target. Detail coefficients of SWT level 4 decomposition (red) and the first derivatives (blue) were prepared for feature extraction.

The schematic plot of the training steps is summarized in Fig. 3. PyTorch (version 1.8.2)^31^ was used in the model development, and we used noise-augmented waveforms with a fixed shape of 2048 × 2 (5.69 s in 360 Hz; two channels) to train the model.

The noise augmentation pipeline consists of two steps. The first step generates various peak shapes and helps the model recognize unseen peaks in other databases (reducing false-negative peaks). We adjusted the frequency of the transformed waveforms and added the minimal Gaussian noise up to ± 10%. These waveforms were multiplied by sinusoidal waveform with amplitudes ranging from 0.5 to 1.5 to represent various peak amplitudes.

In the second step, artificial noises are added with a 50% probability, which helps a model not to classify noises as peaks (reducing false-positive peaks). ECG noise templates of baseline wander, muscle artifact, and electrode motion artifact provided by the MIT-BIH-NST database were used. They were randomly sliced into 2048 pieces and mixed. An estimated amplitude (the difference between the 99.9^th^ percentile and the 0.1^th^ percentile of waveforms) was matched for artificial noises and transformed waves. To reproduce waveforms with various signal-to-noise ratios (SNRs), absolute values of random numbers from a normal distribution with a mean 0 and standard deviation 0.05 less than 2 were multiplied to mixed noises. In testing the MIT-BIH-NST, which used electrode motion artifacts as a noise template, we extracted the unused area of the template.

This noise augmentation generated different waveforms in each epoch, and they were used as model input after flipping or reversing with a 50% probability in a maximum of 100 epochs with 128 mini-batch sizes. The entire portion of the waveform without noise augmentation was used as an internal validation set, and the model performance for the F1 score in this set was monitored to determine early stopping with a patience setting of 10. Adam^32^ and dice loss were selected as the training optimizer and loss function, respectively.

### Peak localization

**Figure 4:**
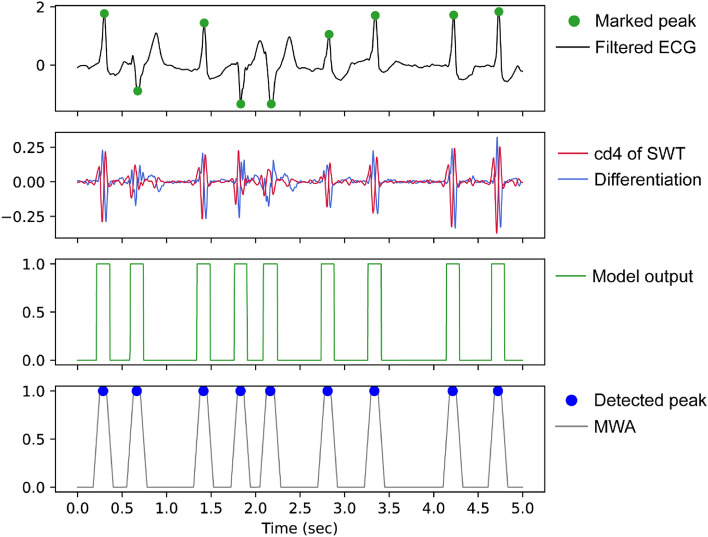
An example of peak localization in patient 114 of the MIT-BIH database. *cd4* detail coefficient of level 4 SWT decomposition; *MWA* moving window average.

The trained model yielded a sequence of probabilities ranging from 0 to 1. We first applied a moving window average, and the sizes of the windows were varied according to the database, 150 ms in the internal validation set and 75 ms in the test set. The sensitivity of the model was slightly decreased in the test set compared with the internal validation set, and we used a smaller size of the moving window to gain higher sensitivity. Next, local maximum peaks higher than 0.5 with a minimal interval of 200 ms (i.e., cardiac refractory period) were defined as a detected position. Figure 4 shows an example of peak localization in a 5-s filtered ECG.

### Testing step

The model performance was evaluated with 3 metrics, such as precision, recall, and F1 score, with 150 ms of tolerance^15^. Cross-database testing was mainly performed in the MIT-BIH, INCART, and QT, while fivefold cross-validation was used to assess the general performance in the totally summarized database. The model was further tested using the MIT-BIH-ST, European ST-T, and TELE databases. In MIT-BIH-NST databases, we changed the noise parameter and evaluated the performance changes.

## Results

### Model performance in MIT-BIH, INCART, and QT

For cross-database validation, we trained the model with one or two databases and tested it with the remaining database. The model performances are summarized in Table 1. The highest model performances in the MIT-BIH, INCART, and QT databases were achieved when the other two databases were used in the training, and their F1 scores were 0.9994, 0.9985, and 0.9999, respectively.

**Table 1:** Model performance according to the training and testing databases.

Testing	Training	TP	FP	FN	Sensitivity	PPV	F1
MIT-BIH	INCART	109,403	90	78	0.9993	0.9992	0.9992
MIT-BIH	QT	109,368	98	113	0.9990	0.9991	0.9990
MIT-BIH	INCART + QT	109,402	61	79	0.9993	0.9994	0.9994
INCART	MIT-BIH	175,561	351	339	0.9981	0.9980	0.9980
INCART	QT	175,469	300	431	0.9975	0.9983	0.9979
INCART	MIT-BIH + QT	175,617	248	283	0.9984	0.9986	0.9985
QT	MIT-BIH	86,989	38	6	0.9999	0.9996	0.9997
QT	INCART	86,980	17	15	0.9998	0.9998	0.9998
QT	MIT-BIH + INCART	86,989	17	6	0.9999	0.9998	0.9999
MIT-BIH + INCART + QT	Other two databases	372,008	326	368	0.9990	0.9991	0.9991
MIT-BIH + INCART + QT	fivefold cross-validation	372,094	262	282	0.9992	0.9993	0.9993

We compared the model performance in the MIT-BIH with previous studies, where SWT was used for peak detection or the cross-database performances were reported (Table 2). The present model achieved the highest F1 scores of 0.9994, which was trailed by 0.9991 in a previous study^13^, while the original study of SWT^7^ showed an F1 score of 0.9986.

**Table 2:** Model performance in the MIT-BIH database by cross-database validation.

Study	Description	Training set	Sensitivity	PPV	F1
Merah et al. 2015^7^	Perhaps SWT	Machine learning not used	0.9984	0.9988	0.9986
Habib et el. 2019^16^	Two-level attention-based 1-D CNN	INCART + QT	0.9780	0.9210	0.9528
Cai et el. 2020^13^	1-D CNN with squeeze-and-excitation networks	CPSC	0.9991	0.9990	0.9991
Vijayarangan et al. 2020^12^	1-D CNN with U-net and inception blocks	CPSC	0.9944	0.9975	0.9965
Zahid et al. 2020^11^	1-D CNN with U-net	CPSC	0.9985	0.9982	0.9983
Ganapathy et al. 2021^8^	1-D CNN with adaptive learning	INCART + TELE	0.9988	0.9996	0.9975
The present study	1-D CNN based on separable convolution	INCART + QT	0.9993	0.9994	0.9994

*CPSC* China Physiological Signal Challenge database^33^, *TELE* Telehealth electrocardiogram recordings^25^.

The sensitivity, PPV and F1 scores of fivefold cross-validation with all three databases were 0.9992, 0.9993 and 0.9993, respectively. When the results of the MIT-BIH predicted by the INCART plus QT, the INCART predicted by the MIT-BIH plus QT, and the QT predicted by the MIT-BIH plus INCART, the evaluation metrics of sensitivity, PPV, and F1 scores were 0.9990, 0.9991, and 0.9991, respectively.

### Effect of preprocessing on the model performance

The F1 score was evaluated when the preprocessing steps were altered during the training process (Table 3). Without SWT, noise filtering, or noise augmentation, the model showed lower performance than the original model with full preprocessing. The difference was maximized in testing the INCART database with no noise filtering.

**Table 3:** F1 scores according to the absence of preprocessing steps in the pipeline.

Testing	Training	Full preprocessing	No SWT	No noise filtering	No noise augmentation
MIT-BIH	INCART	0.9992	0.9990	0.9992	0.9992
MIT-BIH	QT	0.9990	0.9989	0.9989	0.9988
MIT-BIH	INCART + QT	0.9994	0.9992	0.9993	0.9992
INCART	MIT-BIH	0.9980	0.9974	0.9969	0.9979
INCART	QT	0.9979	0.9975	0.9964	0.9971
INCART	MIT-BIH + QT	0.9985	0.9983	0.9970	0.9981
QT	MIT-BIH	0.9997	0.9997	0.9997	0.9997
QT	INCART	0.9998	0.9998	0.9997	0.9998
QT	MIT-BIH + INCART	0.9999	0.9998	0.9999	0.9998

### Model performance in MIT-BIH-ST, European ST-T, and TELE

We used the model trained by all three databases (MIT-BIH, INCART, and QT) to evaluate the MIT-BIH-ST, European ST-T, and TELE databases and F1 scores were 0.9995, 0.9988, and 0.9790, respectively. The summary of the evaluation metrics is presented in Table 4. We compared the F1 scores of the previous studies by performing a multi-database validation with our model and achieved the highest performance on MIT-BIH, INCART, QT, European ST-T, and TELE databases (Table 5).

**Table 4:** The summary of the model performance in MIT-BIH-ST, European ST-T, and TELE databases.

Database	TP	FP	FN	Sensitivity	PPV	F1
MIT-BIH-ST	76,131	39	44	0.9994	0.9995	0.9995
European ST-T	789,364	727	1201	0.9985	0.9991	0.9988
TELE	5849	146	105	0.9824	0.9756	0.9790

**Table 5:** F1 scores of various databases.

Study	MIT-BIH	INCART	QT	MIT-BIH-ST	European-ST	TELE
Belkadi et al. 2021^34^	0.9989				0.9955	
Ganapathy et al. 2021^8^	0.9970	0.9941				0.9585
Rahul et al. 2021^35^	0.9984				0.9975	
Bachi et al. 2020^36^	0.9984		0.9995	0.9948		
Cai et al. 2020^13^	0.9991		0.9995			
Jia et al. 2020^10^	0.9989					0.9725
Smital et al. 2020^37^	0.9986		0.9971		0.9932	
Hibib et al. 2019^16^	0.9486	0.8772	0.9152			
Nayak et al. (1) 2019^38^	0.9994		0.9995			
Nayak et al. (2) 2019^39^	0.9992		0.9997	0.9992		
Nayak et al. (3) 2019^40^	0.9994		0.9997	0.9993	0.9986	
The present study	0.9994	0.9985	0.9999	0.9995	0.9988	0.9790

### Model performance in MIT-BIH-NST

In testing the MIT-BIH-NST database, we used the model trained by the INCART and QTDB databases because the MIT-BIH-NST was generated by adding noises to the MIT-BIH database. The evaluation metrics of sensitivity, PPV, and F1 scores in the MIT-BIH-NST were 0.9568, 0.9441, and 0.9504, respectively, and they were higher than those of the original study that used SWT as the peak enhancement method^7^ (sensitivity, 0.9530; PPV, 0.9398; and F1, 0.9464). The model performance according to different SNRs is summarized in Table 6. The mean F1 score of the SNRs ranging from 24 to 0 was 0.9801, while previous deep learning-based studies with cross-database validation showed mean F1 scores of 0.9685^13^ and 0.9643^12^.

**Table 6:** Model performance in the MIT-BIH-NST database.

Signal-to-noise ratio (dB)	Sensitivity	PPV	F1
24	1.0000	0.9995	0.9998
18	1.0000	0.9993	0.9996
12	0.9998	0.9939	0.9968
6	0.9946	0.9683	0.9813
0	0.9435	0.9037	0.9231
− 6	0.8028	0.8011	0.8020

We explored the changes in the model performance for both the MIT-BIH and MIT-BIH-NST databases according to the degree of noise augmentation in the training process (Table 7). When high noise (high standard deviation in noise amplitudes) is added to the training database, the model performance increases for the MIT-BIH-NST database, while it decreases for the MIT-BIH database. The highest performance for the MIT-BIH-NST was achieved by the standard deviation of 0.5 (F1 score, 0.9759) at the cost of a small performance decrease in the MIT-BIH database (F1 score, 0.9991).

**Table 7:** Model performance in the MIT-BIH-NST database according to the noise amplitudes during training.

Standard deviation of noises	MIT-BIH			MIT-BIH-NST		
	Sensitivity	PPV	F1	Sensitivity	PPV	F1
0.01	0.9993	0.9994	0.9993	0.9551	0.9222	0.9384
0.02	0.9992	0.9995	0.9994	0.9553	0.9158	0.9351
0.05 (present)	0.9993	0.9994	0.9994	0.9568	0.9441	0.9504
0.10	0.9993	0.9994	0.9993	0.9545	0.9528	0.9536
0.20	0.9992	0.9994	0.9993	0.9706	0.9599	0.9652
0.50	0.9988	0.9994	0.9991	0.9754	0.9764	0.9759
1.00	0.9990	0.9990	0.9990	0.9675	0.9758	0.9726

### Examples of false predictions in MIT-BIH database

**Figure 5:**
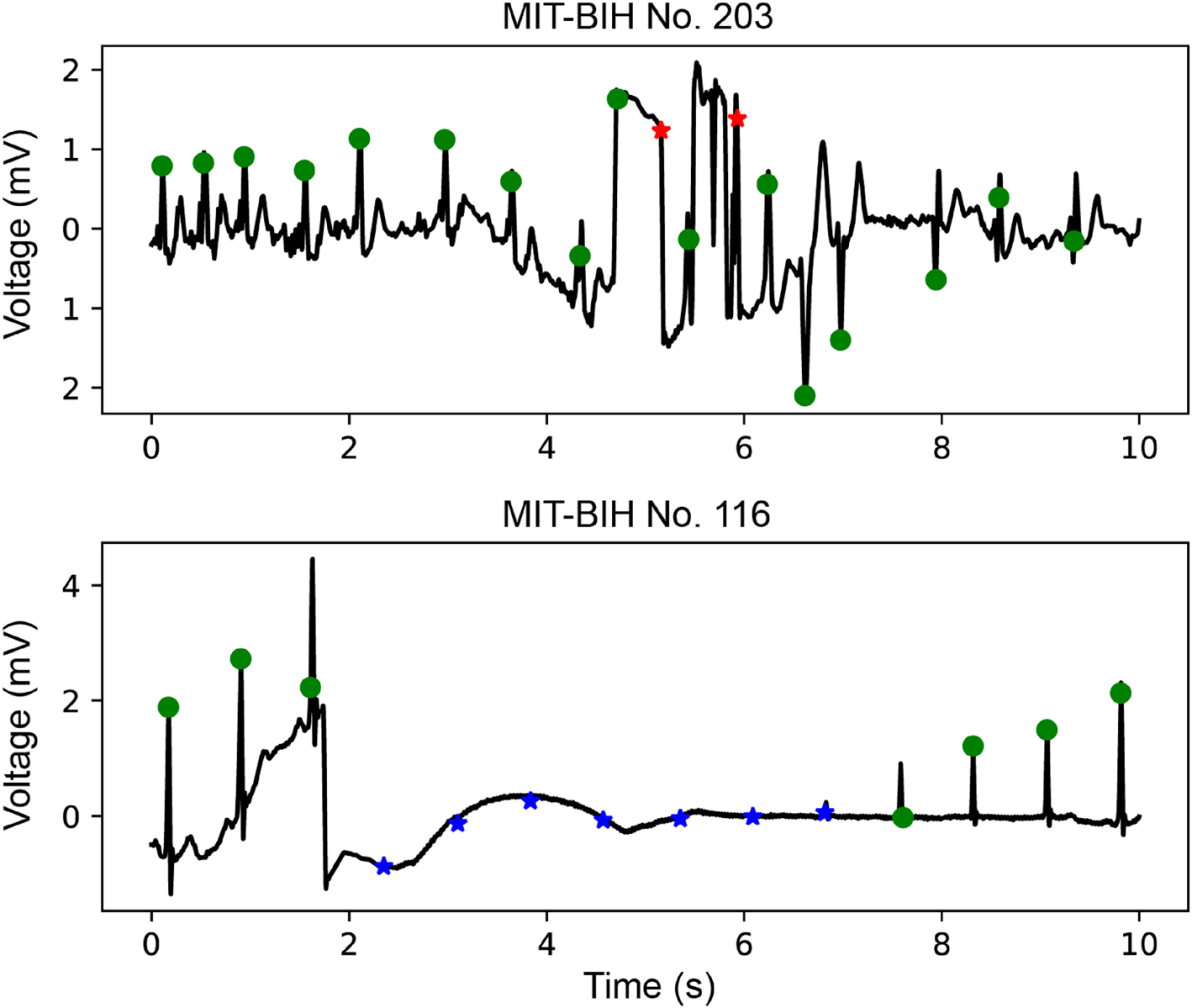
Examples of false predictions from model in MIT-BIH database.

We checked false positive (FP) and false negative (FN) peaks in the MIT-BIH database and reviewed certain cases with a high frequency in FPs (No. 203) and FNs (No. 116) (Fig. 5). The FP peaks in case No. 203 were due to severe noise, which could not be attenuated by the noise-filtering algorithm. Case No. 116 had a nearly flat ECG and detachment of the ECG electrodes was suspected.

## Discussion

The present CNN model was trained with SWT and separable convolution, which enabled a bottleneck-shaped pipeline for peak detection. This state-of-the-art encoder-decoder model achieved robust performance in cross-database validation and showed improved performance when 2 or 3 databases were used during training rather than when 1 database was used. The present model can be applied for different databases with irregular peak shapes and severe noise, and the proposed approach will enhance the handling of long ECG waveforms even from a small number of patients by attenuating the intrinsic difference across databases.

Peak enhancement is a crucial step in conventional peak detection. After extracting peak information from ECG waveforms, a heuristic hyperparameter is mandatory for classifying peaks from noise which is called adaptive thresholding. The Pan-Tompkins algorithm used 0.875 and 0.125 for updating thresholds^1^, and original peak detectors with SWT also used fixed hyperparameters such as 0.25, 0.4, and 0.9^7,17^. Even with a few hyperparameters, the classic peak detector showed favorable results in various databases, and effective peak enhancement methods seemed to significantly contribute to their performance.

Recently, deep learning models with many more hyperparameters than the classic peak detectors have emerged and achieved better performance. Most deep learning-based peak detectors have not utilized preprocessing steps, or have used a minimal peak enhancement, such as differentiation methods. Although a well-structured deep learning model may efficiently extract features from raw databases, overfitting may occur if a small database is used for training. Open source ECG databases have a relatively small number of unique patients compared to the number of peaks; there are more than 100,000 peaks from only 48 patients in the MIT-BIH. In this regard, the model trained by one database may have a different performance in another database^16^.

To solve the above problems, we extract and generalize the peak information before a model training by peak enhancement methods. Peak-enhanced waveforms generated by SWT attenuated the variety of peak shapes based on the different databases and enabled the model to concentrate on the intrinsic features of the peaks, not those of the database. In addition, the noise augmentation technique applied for the present model also contributed to robust training and reduced overfitting.

We adopted the encoder-decoder model based on separable convolution, which has fewer hyperparameters than a conventional convolution but can achieve a comparable performance. The ASPP located at the end of the encoder also provided wide receptive fields. This state-of-the-art deep learning architecture yielded favorable performances in cross-database validations, and the current study may guide the utilization of a database of a small number of patients with long signals.

Cross-database validation is more feasible in real-world applications than κ-fold cross-validation, which produces κ different models. In addition, κ-fold splitting itself may incur data leakage when ECG records from a single database share global features, and its application on other databases may result in an unstable model performance. The pipeline of preprocessing, noise augmentation, model training, and peak localization produced a robust model, and the difference between the fivefold cross-validation and cross-database validation was small (F1 scores 0.9993 vs. 0.9991) in the present study.

Despite these impressive results, there are limitations to be discussed. The model performance may decrease for severe SNRs, and there was a tradeoff phenomenon when we made the model to perform better in noisy ECGs. Further study is warranted to establish a single competent model that shows a favorable response in both clean and noisy ECGs. The present model did not distinguish ventricular flutter from peaks, and this was excluded in the training and testing processes. The present model was established based on 250–360 Hz, and resampling to 360 Hz was mandatory to detect peak positions. It may yield different results when a far different sampling frequency is applied.

## Conclusion

We develop an R-peak detection pipeline with peak enhancement method that includes SWT as an input, and we construct an encoder-decoder architecture based on separable convolution. The model has better performance in open source ECG databases than previous models and also has a successful performance in cross-database validation. The details of the proposed pipeline will be helpful in subsequent studies with a small number of patients.

## Data Availability

The ECGs used in this study were from open source databases and available in https://physionet.org/about/database/ (MIT-BIH, INCART, QT, MIT-BIH-ST, European ST-T, and MIT-BIH-NST databases) and https://dataverse.harvard.edu/ (TELE database). The ECG detection code and the trained model file is also available in Github (https://github.com/dactylogram/ECG_peak_detection). The other data including model training are available from the corresponding author upon request.
